# Clinical trial design in chronic obstructive pulmonary disease: current perspectives and considerations with regard to blinding of tiotropium

**DOI:** 10.1186/1465-9921-13-52

**Published:** 2012-06-22

**Authors:** Kai-Michael Beeh, Jutta Beier, James F Donohue

**Affiliations:** 1 Insaf Respiratory Research Institute, Wiesbaden, Germany; 2 University of North Carolina, Chapel Hill, North Carolina, USA

**Keywords:** Randomisation, Blinded, Blinding, Open-label, Indacaterol, Tiotropium, COPD

## Abstract

Randomised, double-blind, controlled trials are considered the gold standard for evaluating a pharmacological agent, as they minimise any potential bias. However, it is not always possible to perform double-blind trials, particularly for medications delivered via specific devices, e.g. inhalers. In such cases, open-label studies can be employed instead. Methods used to minimise any potential bias introduced by open-label study design include randomisation, crossover study design, and objective measurements of primary efficacy and safety variables. Concise reviews analysing the effect of blinding procedures of comparator drugs on outcomes in respiratory trials are limited. Here, we compare data from different chronic obstructive pulmonary disease trials with once-daily indacaterol versus a blinded or non-blinded comparator. The clinical trial programme for indacaterol, a once-daily, long-acting β_2_-agonist, used tiotropium as a comparator either in an open-label or blinded fashion. Data from these studies showed that the effects of tiotropium were consistent for forced expiratory volume in 1 second, an objective measure, across blinded and non-blinded studies. The data were consistent with previous studies of double-blind tiotropium, suggesting that the open-label use of tiotropium did not introduce treatment bias. The effect of tiotropium on subjective measures (St George’s Respiratory Questionnaire; transition dyspnoea index) varied slightly across blinded and non-blinded studies, indicating that minimal bias was introduced by using open-label tiotropium. Importantly, the studies used randomised, open-label tiotropium patients to treatment allocation, a method shown to minimise bias to a greater degree than blinding. In conclusion, it is important when reporting a clinical trial to be transparent about who was blinded and how the blinding was performed; if the design is open-label, additional efforts must be made to minimise risk of bias. If these recommendations are followed, and the data are considered in the full knowledge of any potential sources of bias, results with tiotropium suggest that data from open-label studies can provide valuable and credible evidence of the effects of therapy.

## Introduction

Randomised, controlled trials (RCTs) are considered the gold standard for evaluating clinical interventions because of their ability to minimise bias [1]. Blinding is an important technique to reduce bias in clinical trials [2,3]. However, it is not always possible to conduct such trials; therefore, alternative study designs are often used.

In this review, we discuss the design of RCTs of inhaled therapies in patients with chronic obstructive pulmonary disease (COPD), with particular regard to the use of blinding and open-label techniques. Data from studies of two once-daily bronchodilators used in COPD, indacaterol and tiotropium, will be used to evaluate the potential for bias in open-label studies.

### Blinding in clinical trials

Administering treatment in a blinded manner is complicated when comparing medications delivered via inhalers of different shapes and sizes [4]. In such cases, it may be necessary to employ more complex methods, such as double-dummy designs. Further difficulties may arise due to the specific properties of some products (e.g. a particular taste or sensation), which may make their identity apparent during administration. The presence of logos on branded products may also present problems if it is not possible to obtain matching placebos with identical branding or to obtain the active drug in an unbranded form. If these difficulties cannot be overcome, it may be necessary to use an open-label design [5]. While double-blind trials are considered as the gold standard for evaluating new products, open-label studies may have some advantages, such as simpler design, lower cost and closer reflection of everyday clinical practice [2,6,7].

In the design of open-label studies, great care is needed to minimise possible sources of bias, which can arise in several ways [3]. For example, patients who know they are receiving an active drug, as opposed to knowing they are on placebo, may report more favourable outcomes because they expect a benefit. The patient’s previous experience of a drug may also affect their reporting of subjective efficacy endpoints or adverse effects. Observers may also be biased by awareness of treatment allocation, which might affect reporting of treatment responses or adverse events. Knowledge of treatment assignment could also affect decisions about remaining on treatment or receiving concomitant medications or other therapy, as well as decisions about inclusion of results in an analysis.

#### *Minimising bias in open-label studies*

Strategies to minimise the likelihood of bias in open-label studies include randomising patients after collection of baseline data [6] and use of crossover study designs [2]. In a crossover design trial, each patient is randomised to a sequence that includes each treatment; hence, each patient can act as their own control for treatment comparisons [2]. However, crossover designs can be limited by the potential for carry-over effects from the previous treatment and administration procedure [2]. The design of crossover studies therefore needs to incorporate appropriate wash-out and familiarisation periods. Other means of minimising bias in open-label studies include basing efficacy outcomes on objective variables [6]. Spirometry is considered the most objective, standardised and reproducible measure of airflow limitation [8], and is therefore unlikely to be subject to bias. Additionally, many clinical trials use a centralised spirometry organisation to further reduce the possibility of variability in the observation. Centralised spirometry may include provision of the same spirometry equipment to all study sites, consistent training of staff who make spirometry assessments and calibration of equipment daily.

### Designing clinical trials of inhaled medications in COPD

Tiotropium is a commonly used once-daily inhaled bronchodilator for the maintenance treatment of patients with COPD and is a key comparator in evaluation of new bronchodilators. However, the use of tiotropium as a comparator presents several difficulties. Firstly, tiotropium is a hygroscopic powder and cannot be removed from the commercial capsules for repackaging into unmarked capsules. Secondly, the commercially available capsules are marked with a logo that can be seen through a window in the inhaler, making it impossible to completely mask the nature of the treatment, and for legal reasons the logo/text cannot be copied onto placebo capsules. The manufacture of tiotropium is also difficult, particularly as the drug is very unstable in the ambient air.

To overcome the problems associated with blinding, a clinical trial of a treatment administered via a dry powder inhaler (DPI) requires considerable resources and possibly assistance from the manufacturer of the comparator DPI to create blinded commercial and placebo products. Ultimately, an alternative clinical trial design may need to be used.

### Experience from the indacaterol clinical trial programme

Three Phase III clinical studies have compared indacaterol with tiotropium. As blinded tiotropium was not available, alternative blinding methods were used (Table 1). The INHANCE study was performed in two stages in an adaptive seamless design [9]. In stage 1, patients were randomised to receive double-blind indacaterol 75, 150, 300, or 600 μg once daily, double-blind formoterol 12 μg twice daily, double-blind placebo, or open-label tiotropium 18 μg once daily for 2 weeks. In stage 2, patients continued treatment with two selected doses of indacaterol (150 or 300 μg, based on 2-week efficacy and safety data), tiotropium, or placebo for 26 weeks, with additional patients recruited and randomised. The INTIME study was a randomised, double-blind, crossover study in which patients received three of the following four treatments, each once-daily for 14 days and each followed by a 14-day washout: double-blind indacaterol 150 or 300 μg, double-blind placebo or third-party blind tiotropium [10]. The blinding of tiotropium treatment was maintained by using a third-party blinding procedure where the study drug was prepared and provided to the patient by persons who were independent of the other clinical trial processes (described in more detail in Table 1). In the 12-week INTENSITY study, a blinded, double-dummy design was used [11]. Following a 2-week run-in, patients were randomised to treatment with indacaterol 150 μg or tiotropium 18 μg, each once-daily for 12 weeks. Patients receiving indacaterol also took a placebo via the inhaler used for tiotropium, and patients receiving tiotropium took a placebo via the inhaler used for indacaterol. Blinding was achieved by specifying that study medications were dispensed by a third party not involved in other aspects of the study. In all three studies, indacaterol was administered via the Breezhaler® DPI device and tiotropium via the Handihaler® DPI device. 

**Table 1:** Description of indacaterol studies using a tiotropium comparator arm*

**Study**	**Description**	**Design**	**Blinding technique and reason**	**Patient entry criteria**	**Study duration**	**Objectives**
INHANCE [9]						
	Randomised, double-blind, placebo-controlled study assessing efficacy, safety and tolerability of two doses of indacaterol in patients with COPD using open-label tiotropium as active control	Double-blind indacaterol and placebo via Breezhaler^®^. Open-label tiotropium via HandiHaler^®^	No placebo to tiotropium was available.* Tiotropium was administered open-label	FEV_1_ <80 and ≥30% predicted FEV_1_/FVC <70%. Smoking history ≥20 pack-years	26 weeks	First objective: superiority of indacaterol to placebo using trough FEV_1_ at 12 weeks. Second objective: non-inferiority of indacaterol to tiotropium using trough FEV_1_ at 12 weeks
INTIME [10]	Randomised, blinded, placebo-controlled, multicentre, three-period, incomplete block, multi-dose crossover study to determine the effect on lung function of indacaterol in patients with moderate-to-severe COPD, using tiotropium as an active control	Double-blind indacaterol and placebo via Breezhaler^®^. Third-party blinding of tiotropium (via HandiHaler^®^)^†^	Indacaterol and its matching placebo were made identical in appearance and were dispensed in such a manner to make them indistinguishable to patients and all blinded study personnel. An exact physical match to tiotropium was not available. Blinding of tiotropium was maintained by use of third-party blinding procedures^†^	FEV_1_ <80 and ≥30% predicted FEV_1_/FVC <70%. Smoking history ≥10 pack-years	70 days (14 days treatment with four different treatments in three separate periods with a washout of 14 days between treatments)	First objective: superiority of indacaterol to placebo using trough FEV_1_ at 14 days. Second objective: non-inferiority of indacaterol to tiotropium using trough FEV_1_ at 14 days
INTENSITY [11]	Randomised, parallel-group, blinded, double-dummy study to compare the efficacy and safety of indacaterol delivered via a SDDPI with tiotropium delivered via a HandiHaler^®^ in patients with moderate-to-severe COPD	Blind, double-dummy indacaterol via Breezhaler^®^ versus tiotropium via HandiHaler^®^	Patients receiving indacaterol also took placebo via the inhaler used for tiotropium, and patients receiving tiotropium took placebo via the inhaler used for indacaterol. The colour of the capsules was compatible, but the placebo did not have the markings. The blinding of tiotropium was maintained by use of an unblinded individual, who was not involved in any study assessments, to administer treatment. Patients and investigators therefore remained blind to treatment allocation	FEV_1_ <80 and ≥30% predicted FEV_1_/FVC <70%. Smoking history ≥10 pack-years	12 weeks	First objective: non-inferiority of indacaterol to tiotropium using trough FEV_1_ at 12 weeks Second objective: superiority of indacaterol to tiotropium using trough FEV_1_ at 12 weeks

*The blinding of tiotropium is complicated by the fact that patients place a capsule in the inhalation device (HandiHaler®) and are able to see the logo on the capsule through a window in the device. Neither placebo tiotropium capsules with such a logo nor active tiotropium capsules without such a logo could be obtained for use in these studies of indacaterol; therefore, it was not possible to conduct traditionally designed double-blind studies.

^†^Study drug was prepared and provided to the patient each morning, either at home or in the clinic by persons who were independent of the other clinical trial processes (referred to as ‘independent study blinding co-ordinators’, ISBCs) to preserve the integrity of the blind. Two ISBCs were required for each daily study drug administration to each patient. The first (non-blinded) ISBC (who had no contact with the patient) prepared the study drugs and devices. The second (blinded) ISBC provided the patient with the prepared study drug and devices, monitored administration of the drug by patients and ensured that the blinding was maintained throughout. Both ISBCs completed the third-party blinding log for every drug administration, to provide evidence that the blinding procedure was strictly followed.

COPD: chronic obstructive pulmonary disease; FEV_1_: forced expiratory volume in 1 second; FVC: forced vital capacity; SDDPI: single dose dry powder inhaler.

Patient populations were similar across the three studies, which enrolled patients with moderate-to-severe COPD (forced expiratory volume in 1 second [FEV_1_ <80% and ≥30% predicted; FEV_1_/forced vital capacity [FVC] <0.7) [12-14], and a smoking history of ≥10 pack-years (INTIME and INTENSITY [10,11]) or ≥20 pack-years (INHANCE [9]). The primary endpoint was trough FEV_1_ (an objective endpoint) in all three studies, with the primary comparisons being indacaterol versus placebo at Week 12 in INHANCE [9], Week 2 in INTIME [10] and indacaterol versus tiotropium at Week 12 in INTENSITY [11].

#### *Effect of study blinding on objective measures: lung function*

The results of 24-hours post-dose (trough) FEV_1_ for active treatment versus placebo in the INHANCE and INTIME studies are shown in Table 2. The treatment differences for tiotropium versus placebo were similar across both studies, even though they differed in blinding of the tiotropium arm [9,10]. Treatment differences for indacaterol versus placebo were also consistent across both studies. 

**Table 2:** Key results from indacaterol studies with a tiotropium comparator arm and double-blind studies with tiotropium as the primary treatment of interest

**Study**	**Design**	**Patient characteristics**	**Time**	**Trough FEV**_**1**_**versus placebo (mL)**	**Proportion of patients achieving MCID**	
					**SGRQ (%)**	**TDI (%)**
***Indacaterol versus tiotropium studies***						
INHANCE [9]	OL*	FEV_1_ <80% and ≥30% predicted; FEV_1_/FVC <0.7; Mean FEV_1_ % predicted^¶^: Indacaterol 150 μg 56.1, Indacaterol 300 μg 56.3, Tiotropium 53.9, Placebo 56.1	12 wk	Tiotropium: 140	44.9^†^	55.0^†^
				Indacaterol 150 μg: 180	51.9^†^	58.9^†^
				Indacaterol 300 μg: 180	50.1^†^	65.8^†^
			26 wk	Tiotropium: 140	47.3	57.3
				Indacaterol 150 μg: 160	57.8	62.4
				Indacaterol 300 μg: 180	52.5	70.8
INTIME [10]	TPB	FEV_1_ <80 and ≥30% predicted; FEV_1_/FVC <0.7; Mean FEV_1_ % predicted^¶^ 56.7	2 wk	Tiotropium: 120	—	—
				Indacaterol 150 μg: 170	—	—
				Indacaterol 300 μg: 150	—	—
INTENSITY [11]	B	FEV_1_ <80 and ≥30% predicted; FEV_1_/FVC <0.7; Mean FEV_1_ % predicted^¶^: Indacaterol 54.6, Tiotropium 54.3	12 wk	Tiotropium: —	42.5	50.1
				Indacaterol 150 μg	50.5	57.9
***Tiotropium versus placebo studies***						
Beeh et al. 2006 [15]	DB	FEV_1_ ≤70% predicted; FEV_1_/FVC <0.7^‡^; Mean FEV_1_ % predicted^‡^: Total 45.4, Tiotropium 45.3, Placebo 45.7	12 wk	79	—	—
Freeman et al. 2007 [16]	DB	FEV_1_ 30–65% predicted; FEV_1_/FVC <0.7^§^; Mean FEV_1_ % predicted^‡^: Total 48.9, Tiotropium 47.9, Placebo 49.9	12 wk	60	—	—
Johansson et al. 2008 [17]	DB	FEV_1_ >60% predicted; FEV_1_/FVC <0.7^¶^; Mean FEV_1_ % predicted^§^: Tiotropium 73.6, Placebo 73.2	12 wk	118	—	—
Moita et al. 2008 [18]	DB	FEV_1_ ≤70% predicted; FEV_1_/FVC <0.7^‡^; Mean FEV_1_ % predicted^‡^: Tiotropium: non-smokers 38.4, smokers 44.4; Placebo: non-smokers 42.3, smokers 40.4	12 wk	102	—	—
Verkindre et al. 2006 [19]	DB	FEV_1_ ≤50% predicted; FEV_1_/SVC ≤0.7; lung hyperinflation^‡^; Mean FEV_1_ % predicted^‡^: Tiotropium 34.7, Placebo 35.8	12 wk	110	59	—
Niewoehner et al. 2005 [20]	DB	FEV_1_ ≤60% predicted; FEV_1_/FVC <0.7^‡^; Mean FEV_1_ % predicted^‡^: Tiotropium 35.6, Placebo 35.6	13 wk	100	—	—
			26 wk	100	—	—
Brusasco et al. 2003 [21]	DB	FEV_1_ ≤65% predicted; FEV_1_/FVC <0.7^‡^; Mean FEV_1_ % predicted^‡^: Tiotropium 39.2, Placebo 38.7	26 wk	120	48.9	43.1
Tonnel et al. 2008 [22]	DB	FEV_1_ 20–70% predicted; FEV_1_/FVC 0.7^#^; Mean FEV_1_ % predicted^‡^: Tiotropium 47.5, Placebo 46.2	12 wk	—	60^||^	—
			26 wk	—	60^||^	—
			39 wk	100	59.1	—
Chan et al. 2007 [23]	DB	FEV_1_ ≤65% predicted; FEV_1_/FVC <0.7^‡^; Mean FEV_1_ % predicted^‡^: Tiotropium 39.4, Placebo 39.3	11 wk	100^||^	—	—
			48 wk	100	53	—
Casaburi et al. 2002 [24]	DB	FEV_1_ ≤65% predicted; FEV_1_/FVC <0.7^‡^; Mean FEV_1_ % predicted^‡^: Tiotropium 39.1, Placebo 38.1	13 wk	148^||^	—	42–47^≈^
			25 wk	148^||^	—	42–47^≈^
			1 yr	150^||^	49	47^≈^
Tashkin et al. 2008 [25]	DB	FEV_1_ ≤70% predicted; FEV_1_/FVC <0.7^¶^; Mean FEV_1_ % predicted^¶^: Tiotropium 47.7, Placebo 47.4	26 wk	100^||^	—	—
			4 yr	87–103	45%	—

Data are least square mean for INHANCE, INTIME and INTENSITY; other publications did not describe the type of mean.

*Tiotropium arm (indacaterol and placebo were DB).

^†^Data on file at Novartis.

^‡^Not stated whether pre- or post-bronchodilator.

^§^Pre-bronchodilator.

^S¶^Post-bronchodilator.

^#^Pre- and post-bronchodilator.

^||^Estimated from a figure.

^≈^Values are given as 42–47% across all timepoints up to 2 years (greatest improvement versus baseline was seen at 2 years).

Definitions of trough FEV_1_ varied (mean of 23 hours 10 minutes and 23 hours 45 minutes post-dose for INHANCE, INTIME and INTENSITY, Beeh et al. 2006 [15], Verkindre et al. 2006 [19] and Brusasco et al. 2003 [21]; 10 minutes pre-dose for Freeman et al. 2007 [16], Johansson et al. 2008 [17] and Chan et al. 2007 [23]; 30 minutes pre-dose for Tonnel et al. 2008 [22]; 1 hour pre-dose for Casaburi et al. 2002 [24]; and ‘pre-dose’ for Niewoehner et al. 2005 [20] and Tashkin et al. 2008 [25]. All studies recruited patients aged ≥40 years with a smoking history of ≥10 pack-years (≥20 years for INHANCE).

MCID: minimum clinically important difference; SGRQ: St. George’s Respiratory Questionnaire; TDI: transition dyspnoea index; OL: open-label; FEV_1_: forced expiratory volume in 1 second; FVC: forced vital capacity; TPB: third-party blinding; B: blinded; DB: double-blind; SVC: slow vital capacity.

Table 2 also shows FEV_1_ data from previous studies in which tiotropium was the primary treatment of interest. It should be noted that there were differences in entry criteria and in definitions of trough FEV_1_ between studies.

**Figure 1:**
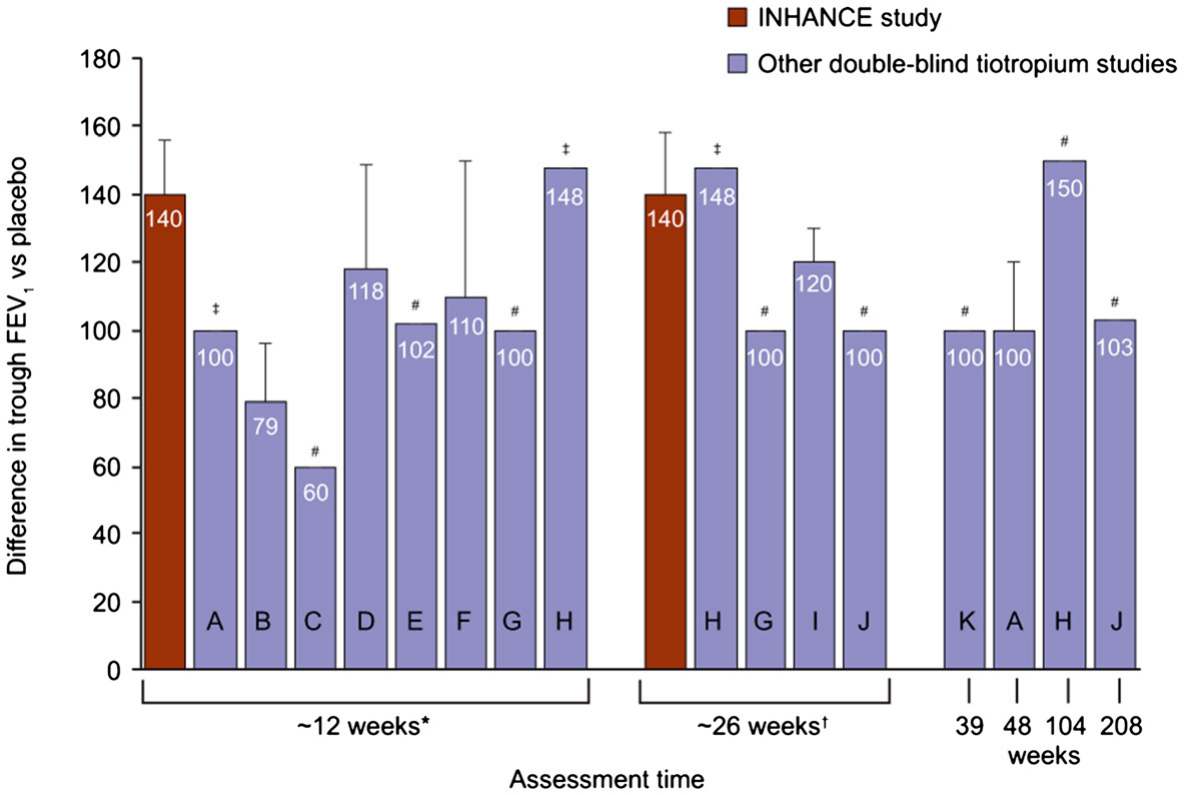
**Differences in trough forced expiratory volume in 1 second (FEV**_**1**_**) between tiotropium and placebo in INHANCE**[9]**and studies in which tiotropium was the primary treatment of investigation.** Values are means and standard errors. *Includes assessments made at 11, 12 and 13 weeks; ^†^Includes assessments made at 25 weeks; ^‡^Estimated from a figure (no standard error available); ^#^No standard error available.

In the INHANCE study, the difference in trough FEV_1_ between open-label tiotropium and placebo was 140 mL at both Weeks 12 and 26 [9]. These values are similar to those recorded during the previous double-blind studies of tiotropium at 12 weeks (60–148 mL), 26 weeks (100–148 mL) and over the longer term (100–150 mL; Table 2 and Figure 1). 

These findings suggest that there was no bias against tiotropium for FEV_1_ in the open-label INHANCE study. The 140 mL difference between tiotropium and placebo in the indacaterol studies was greater than the differences seen at 12 and 26 weeks in the majority of the double-blind studies of tiotropium, raising the possibility that the bias, if any, was favouring the performance of tiotropium (Figure 1). However, the variation in FEV_1_ even between the previous double-blind studies may partly reflect differences in enrolment criteria, such as COPD severity or patient characteristics.

In the INTENSITY study, which did not include a placebo arm, trough FEV_1_ at Week 12 was similar with indacaterol (144 mL) and tiotropium (143 mL), and non-inferiority was demonstrated [11]. Mean changes from baseline in trough FEV_1_ were also similar between indacaterol (130 mL) and tiotropium (120 mL) in that study.

#### *Effect of study blinding on subjective measures: health-related quality of life*

As well as FEV_1_, many regulatory authorities recommend that clinical trials in COPD include an endpoint that reflects clinical benefit of treatment using a validated measure, such as the St. George’s Respiratory Questionnaire (SGRQ) [26,27].

The SGRQ is used to assess health status in patients with chronic respiratory disease and was included as a measure in the INHANCE and INTENSITY studies. The questionnaire, which is completed by the patient, comprises of 50 questions in three domains: symptoms, activity (limitations) and impacts (of disease), which are calculated to provide a score between 0 (best) and 100 (worst). The minimal clinically important difference (MCID) for SGRQ score that is generally accepted as indicating an improvement over placebo or from baseline is −4 units [28].

**Figure 2:**
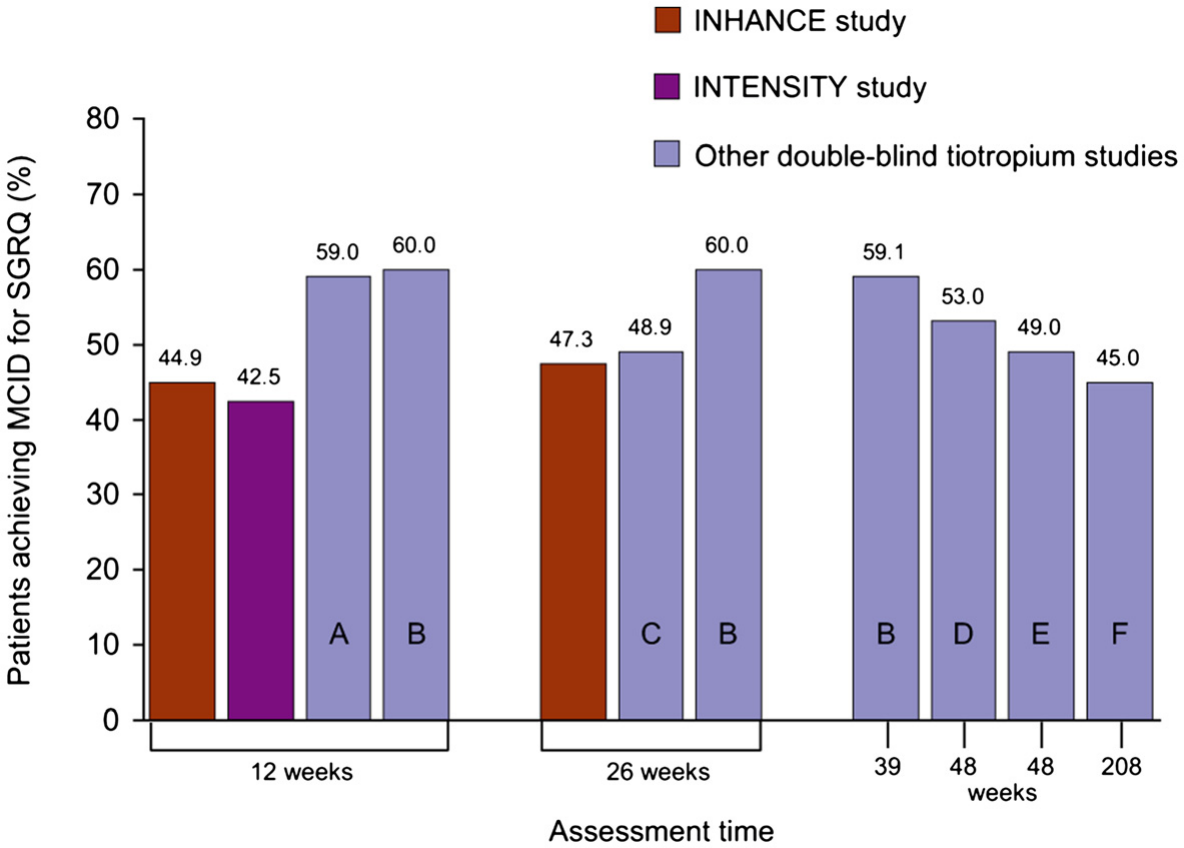
**Percentages of tiotropium-treated patients achieving the minimum clinically important difference (MCID) for St. George’s Respiratory Questionnaire (SGRQ) score in INHANCE**[29]**, INTENSITY**[11]**and other studies in which tiotropium was the primary treatment of investigation.**

After 12 weeks of treatment, the MCID for change in SGRQ from baseline was achieved by 44.9% of tiotropium-treated patients (with a mean improvement of −1.1 units) in the INHANCE study (open-label tiotropium) and 42.5% of patients (with a mean improvement of −3.0 units) in the INTENSITY study (blinded tiotropium) [11,29] (Table 2). These values are slightly lower than those recorded at 12 weeks in studies where tiotropium was the primary treatment of interest (Figure 2). 

At 26 weeks, 47.3% of tiotropium-treated patients in INHANCE achieved the MCID for change in SGRQ (with a mean improvement of −1.0 units) compared with a range of 49–60% in published tiotropium studies. Overall, the percentage of patients treated with open-label tiotropium in INHANCE who achieved the MCID for change in SGRQ at 12 and 26 weeks, were within the range of values recorded across all of the double-blind tiotropium studies over the durations of 12 weeks to 4 years (Table 2 and Figure 2). Again, it should be noted that, even in double-blind studies of tiotropium, there was variability in the proportion of patients who achieved the MCID for SGRQ (and in the mean improvement scores; –2.7 to −6.5).

These findings indicate that minimal bias was introduced with the open-label design of the tiotropium comparator arm in the INHANCE study.

#### *Dyspnoea – transition dyspnoea index (TDI)*

Dyspnoea, or breathlessness, is the major limiting symptom for COPD patients and is often measured using the TDI [30], a tool recommended by regulatory authorities for inclusion in clinical trials of treatments for COPD [26].

The TDI is a multidimensional instrument that measures change from the baseline dyspnoea index (BDI) over time [31]. The BDI considers three components (functional impairment, magnitude of task and magnitude of effort), each rated from 0 (severe dyspnoea) to 4 (no dyspnoea). The TDI measures changes from baseline in each domain of the BDI on a scale of +3 (major improvement) to −3 (major deterioration) and has been shown to be valid, reliable and responsive [31,32]. The MCID for the TDI is an improvement from the BDI score of ≥1 unit [33].

**Figure 3:**
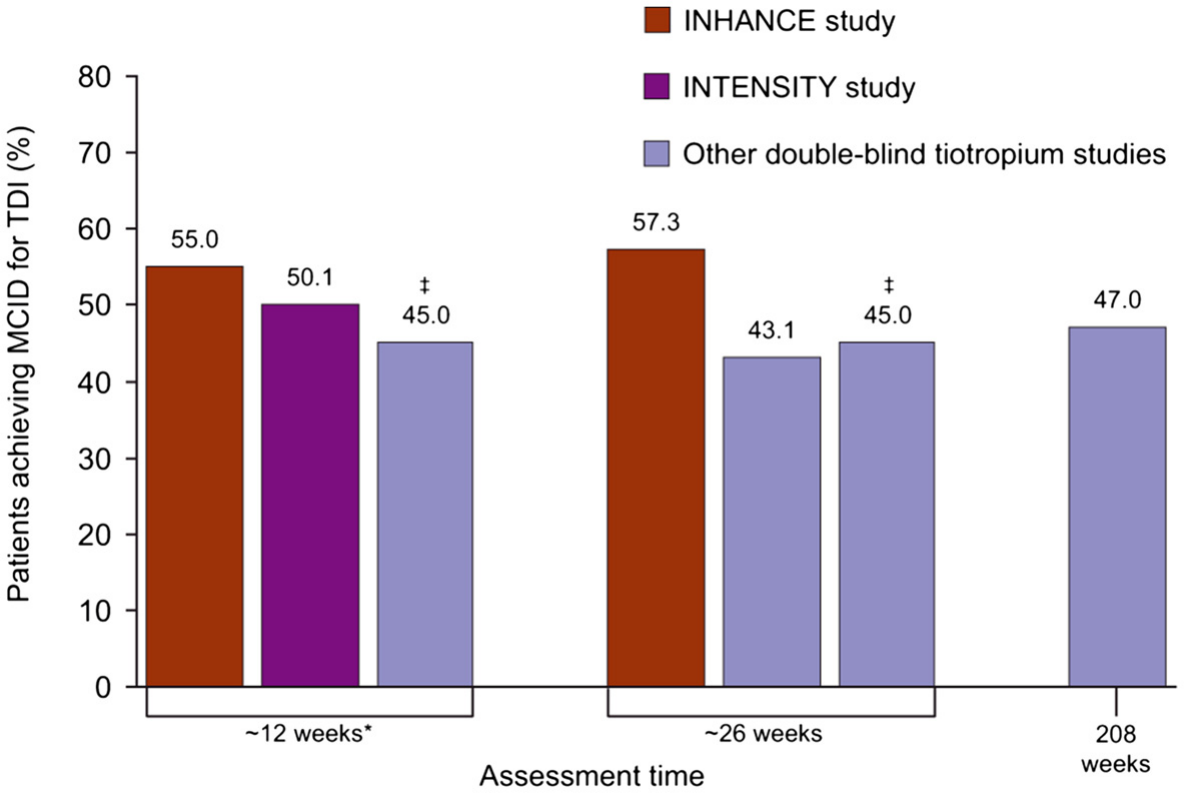
**Percentages of tiotropium-treated patients achieving the minimum clinically important difference (MCID) for transition dyspnoea index (TDI) in INHANCE**[9]**, INTENSITY**[11]**and other studies in which tiotropium was the primary treatment of investigation.** *Includes assessments made at 13 weeks; ^†^Includes assessments made at 25 weeks; ^‡^Precise value is not given (values stated as range of 42–47% across all timepoints).

The proportion of patients achieving an improvement in TDI equal or greater to the MCID was assessed in the INHANCE and INTENSITY trials of indacaterol (Table 2). In the INHANCE study (open-label tiotropium), the MCID for TDI was achieved by 55.0% of tiotropium-treated patients at 12 weeks (with a mean improvement of 0.75 points) and 57.3% at 26 weeks (with a mean improvement of 0.87 points) [9], while in the INTENSITY study (blinded tiotropium), 50.1% of tiotropium-treated patients achieved the MCID at 12 weeks (with a mean improvement of 1.43 points) [11]. These values are slightly higher than the values recorded during the previous studies of tiotropium [21,24], again demonstrating that the open-label design of INHANCE did not result in a bias against tiotropium (mean improvement of 0.8–1.28 points) (Table 2 and Figure 3). 

#### *Adverse events*

In the INHANCE study, the overall incidence of adverse events and the most commonly occurring adverse events were similar across the treatment groups (indacaterol 150 or 300 μg, tiotropium, or placebo) [9]. Respiratory tract infections and respiratory events were the most common type of adverse events, serious events, and those leading to withdrawal of study treatment. In the INTIME study, the overall incidence of adverse events was similar across all treatments (indacaterol 150 or 300 μg, tiotropium or placebo), and these were predominantly mild or moderate in severity [10]. The most frequent adverse events were cough, COPD exacerbation, and nasopharyngitis. In the INTENSITY study, adverse events were reported for similar proportions of patients in the two treatment groups (indacaterol and tiotropium), with the most common events generally reflecting the typical disease characteristics of COPD [11]. These results reflect adverse events observed in previous double-blind trials of tiotropium compared with placebo [17,18,22,24,25].

### Additional evidence on the effect of study blinding on treatment effect

It is worth noting that the third-party blinding procedure used in the INTIME study required administration of the study drug to the patient by a blinded individual, and therefore the forced high compliance may potentially bias results over what might be seen in a more traditional clinical trial. In addition, the high manpower intensity required for third-party blinding may limit these studies to short periods of time.

Previous reports on the effects of blinding in RCTs have been conflicting. Several studies examining the effects of differing blinding techniques in RCTs from a range of therapy areas have found that open-label studies tend to exaggerate the benefits of treatment when they are included in meta-analyses [34-37]. For example, Schulz et al*.* found that in open-label RCTs, odds ratios were exaggerated by 17% [34]. Other studies of RCT blinding have found that an open-label trial design is not associated with treatment bias [38-40]. Moreover, investigations have shown that the adequacy of randomisation has a greater influence on treatment bias than blinding [39].

Observational studies are considered inherently biased as they are both open-label and non-randomised [41,42]. However, a comparison of treatment bias in observational studies versus RCTs found that in 17 out of 19 analyses, the estimates of treatment effects from observational studies were similar to those from RCTs; in only two of the 19 analyses did the combined magnitude of the treatment effect in the observational studies lie outside the 95% confidence interval for the combined magnitude in the RCTs [43]. Data from epidemiological studies, while very different in design and quality from a RCT indicate that objective outcomes are less affected by bias associated with an open-label study design than subjective outcomes [44]. Thus, even under conditions where bias is expected, an open-label study design need not introduce bias, particularly if assessments are based on objective measurements.

## Conclusions

Double-blind RCTs remain the gold standard for evaluating interventions in chronic diseases such as COPD. However, many drugs in COPD, particularly bronchodilators, have an acute effect on both subjective and objective outcomes, therefore full blinding cannot be guaranteed. If an open-label design is chosen, the potential for bias must be taken into account and the selection of outcome measures is of pivotal importance. Measures such as airway function are considered objective and less prone to bias, and are therefore recommended as primary endpoints. Despite possible limitations associated with functional endpoints such as FEV_1_ in COPD, these variables have proven reliable, are subject to little or no placebo effect, and have produced consistent results that are largely independent of study population, treatment duration and differences in study design and blinding.

This low likelihood of bias with objective measures of airway function is also supported by the fact that spirometry has rigorous standards for performance in clinical trials; any incomplete effort that can introduce bias is detected by the technician/central agency and not included. Thus, selection of an objective outcome measure such as FEV_1_ in an open-label study in COPD is unlikely to introduce relevant bias if technical requirements are met and established guidelines are followed [45].

While this holds true for FEV_1_ and, potentially, other objective endpoints in COPD, the inclusion of more subjective, patient-reported outcomes may pose a larger challenge. However, data from studies comparing indacaterol with tiotropium demonstrate that the effects of tiotropium on subjective measures (SGRQ; TDI) vary slightly across blinded and non-blinded studies, indicating that minimal bias was introduced by using open-label tiotropium. It is likely that the appropriate use of randomisation in the comparative studies was an important factor in minimising potential bias. Nevertheless, the extent to which instruments such as symptom indices or health status questionnaires are affected by blinding issues in the absence of a placebo control, are as yet poorly characterised or unknown. Minimum clinically important differences for these instruments have been established using well-controlled, blinded trials against a placebo comparator, thus hampering the clinical interpretation of data generated in open-label studies. As more open-label data on these outcomes become available in the future, this may help to clarify the clinical significance of treatment-associated changes observed in the open-label studies discussed above.

In conclusion, when reporting a clinical trial, it is important to be transparent about who exactly was blinded and how. If an open-label design is necessary, additional efforts should be made to minimise the risk of bias. If these recommendations are followed, and the data are considered in the full knowledge of any potential sources of bias, results with tiotropium suggest that data from open-label studies can provide valuable and credible evidence of the effects of a therapy.

## Abbreviations

B, Blinded; BDI, Baseline dyspnoea index; COPD, Chronic obstructive pulmonary disease; DB, Double-blind; DPI, Dry powder inhaler; FEV_1_, Forced expiratory volume in 1 second; FVC, Forced vital capacity; ISBC, Independent study blinding co-ordinators; MCID, Minimal clinically important difference; OL, Open-label; RCTs, Randomised, controlled trials; SDDPI, Single dose dry powder inhaler; SGRQ, St. George’s Respiratory Questionnaire; SVC, Slow vital capacity; TDI, Transition dyspnoea index; TPB, Third-party blinding.

## Competing interests

K-MB and JB have received compensation for organising or participating in advisory boards for Cytos, Boehringer Ingelheim, AstraZeneca, Novartis and Revotar Biopharmaceuticals. K-MB has participated as a speaker in scientific meetings or courses organised and financed by various pharmaceutical companies (AstraZeneca, Boehringer, Novartis, Pfizer and Takeda) in the past 5 years. The institution where K-MB and JB are currently employed has received compensations for the design, performance or participation in single or multicentre clinical trials in the past 5 years from several companies including Almirall Prodesfarma, Altana, AstraZeneca, Boehringer Ingelheim, Cytos, GSK, Medapharma, Merck Sharp & Dohme, Mundipharma, Novartis, Pfizer and Revotar Biopharmaceuticals.

JFD has served as a consultant and advisor and performed studies under contract for Novartis and Boehringer Ingelheim.

## Authors’ contributions

K-MB, JB and JFD conceived the review and participated in all stages of developing the manuscript. All authors have read and approved the final manuscript.
